# A blood-based biomarker panel to risk-stratify mild traumatic brain injury

**DOI:** 10.1371/journal.pone.0173798

**Published:** 2017-03-29

**Authors:** Richa Sharma, Alexandra Rosenberg, Ellen R. Bennett, Daniel T. Laskowitz, Shawn K. Acheson

**Affiliations:** 1 School of Medicine, Duke University Medical Center, Durham, North Carolina, United States of America; 2 Department of Neurology, Duke University Medical Center, Durham, North Carolina, United States of America; 3 Durham VA Medical Center, Durham, North Carolina, United States of America; 4 Department of Psychiatry and Behavioral Sciences, Duke University Medical Center, Durham, North Carolina, United States of America; University of Florida, UNITED STATES

## Abstract

Mild traumatic brain injury (TBI) accounts for the vast majority of the nearly two million brain injuries suffered in the United States each year. Mild TBI is commonly classified as complicated (radiographic evidence of intracranial injury) or uncomplicated (radiographically negative). Such a distinction is important because it helps to determine the need for further neuroimaging, potential admission, or neurosurgical intervention. Unfortunately, imaging modalities such as computed tomography (CT) and magnetic resonance imaging (MRI) are costly and not without some risk. The purpose of this study was to screen 87 serum biomarkers to identify a select panel of biomarkers that would predict the presence of intracranial injury as determined by initial brain CT. Serum was collected from 110 patients who sustained a mild TBI within 24 hours of blood draw. Two models were created. In the broad inclusive model, 72kDa type IV collagenase (MMP-2), C-reactive protein (CRP), creatine kinase B type (CKBB), fatty acid binding protein—heart (hFABP), granulocyte-macrophage colony-stimulating factor (GM-CSF) and malondialdehyde modified low density lipoprotein (MDA-LDL) significantly predicted injury visualized on CT, yielding an overall c-statistic of 0.975 and a negative predictive value (NPV) of 98.6. In the parsimonious model, MMP-2, CRP, and CKBB type significantly predicted injury visualized on CT, yielding an overall c-statistic of 0.964 and a negative predictive value (NPV) of 97.2. These results suggest that a serum based biomarker panel can accurately differentiate patients with complicated mild TBI from those with uncomplicated mild TBI. Such a panel could be useful to guide early triage decisions, including the need for further evaluation or admission, especially in those environments in which resources are limited.

## Introduction

### Background

Traumatic brain injury (TBI) is a major cause of morbidity and mortality globally. In the United States alone, approximately 1.7 million people suffer from TBIs annually [1], and globally, TBI is the leading cause of death and disability in children and young adults [2]. TBI is graded by severity, including designations of mild, moderate, and severe based on Glasgow Coma Score (GCS) [3]. Mild TBI (mTBI), defined as presentation GCS 13–15, is the most common type of TBI, constituting nearly 70–90% of treated brain injuries in the US alone.

The morbidity associated with TBI (even mTBI) is considerable. Studies have shown that between 1 to 20 percent of patients with mTBI develop persistent physical, cognitive, and behavioral impairments [4, 5].

Upon initial clinical presentation, patients must be carefully evaluated for potential intracranial complications, which, although relatively infrequent, require neurosurgical intervention in a minority of cases (0.4%-1.0%; [6]). Clinical assessment of patients with mTBI may be challenging, as the absence of focal neurological findings on initial clinical exam does not preclude potentially serious intracranial injury. Thus, imaging studies such as brain CT are routinely performed. However, performing routine head CT’s on all patients is resource-intensive, and results in unnecessary radiation exposure given the proportion of patients that ultimately require neurosurgical invention. Unnecessary radiation exposure can be particularly problematic in the pediatric population. Moreover, CT may not be readily available in rural, military, or sports field settings. In such settings, a rapid decision regarding potential risk in the face of inadequate information must be made when considering early routing or transfer to a medical center equipped to deal with more severe injuries.

Several clinical decision-making rules have been developed to address this dilemma. These include the Canadian CT Head Rule and Neurotraumatology Committee of the World Federation of Neurosurgical Societies, the New Orleans Criteria, the National Emergency X-Radiography Utilization Study II (NEXUS-II), the National Institute of Clinical Excellence guideline, and the Scandinavian Neurotrauma Committee guidelines. In general, these clinical decision-making guidelines incorporate clinical and historical criteria, such has the presence and duration of loss of consciousness, retrograde amnesia, mechanism of injury, seizure, age, headache, or vomiting. Although several of these algorithms such as the Canadian CT Head Rule (CCTHR) and New Orleans criteria (NOC) have been demonstrated to have high sensitivity, they have relatively poor specificity [6], and for a variety of reasons, these algorithms are infrequently used clinically [7]. Thus, wide variability still exists with regard to initial treatment of patients with mTBI as physicians must weigh the risk/benefit ratio of whether a CT is appropriate considering issues of cost, radiation exposure, and the need for transfer. This unmet clinical need is emphasized in a recent policy statement by the American College of Emergency Physicians, which stated that more conclusive evidence is needed to help identify in a timely manner the small but important number of patients who may have intracranial pathology despite a normal neurological exam and minimal initial symptoms [8].

Given these limitations of the clinical examination, some have argued that a very low threshold should be used for obtaining a CT scan in individuals presenting with a closed head injury. However, more than one million individuals present to Emergency Departments annually and are seen and released without requiring hospitalization. The use of imaging as a stand-alone marker for intracranial pathology in patients with closed head injuries would lead to unnecessary interventions and elevated cost for patients and hospitals. For example, between 1992 and 2000, there was a notable increase in the rate of utilization of head CT scans by Canadian emergency departments for assessment of TBI, but only 10% of these scans showed clinically relevant intracranial pathology [9]. Among patients classified as mTBI, only 1% required neurosurgical intervention. The cost of a non-contrasted head CT in a US emergency department varies from $391.62 to $2,015 [10]. In addition to monetary ramifications of unnecessary head CTs, CT scans can lead to treatment of incidental findings and the harm of exposure to ionizing radiation. However, given the absence of other diagnostic markers, the demand for head CT is high. The supply of CT scanners, though widely available in the US, can be limited in certain American emergency departments including in smaller, rural, and critical access hospitals [11]. The availability of these scanners drops precipitously worldwide in resource-limited countries and war zones. This supply-demand mismatch suggests a need for a sensitive, point-of-care screening tool with the ability to determine risk of significant brain injury to minimize unnecessary imaging.

The initial management of patients with mTBI would be greatly enhanced by rapid and widely available approaches that provide real-time diagnostic information to complement the clinical exam. One promising approach would be to establish a biomarker-based diagnostic to identify patients at high risk for intracranial injury. This has been an area of clinical research focus for our group, and we have identified a series of biomarkers that are predictive of ischemic stroke, intracranial hemorrhage, and delayed ischemic deficit in subarachnoid hemorrhage [12–14]. These biomarkers include surrogates of gliosis and neuroinflammation, such as S100B, glial fibrillary acidic protein (GFAP), monocyte chemoattractant protein–1 (MCP-1) [14–16]; acute inflammatory responses, such as C-reactive protein (CRP), MMP-9, and D-dimer [12, 15, 16]; and, neuronal stress such as beta-nerve growth factor (BNGF) [14].

Indeed, there have been multiple biomarkers of TBI proposed in the literature, ranging from markers of inflammation such as tumor necrosis factor—alpha (TNF-α) [17–20] and interleukin 1 beta (IL-1 β) [21, 22], to markers of astrocytic activation such as S100B [23–25]. Levels of markers of neuronal injury such as αΑ-II spectrin breakdown products correlate with severe TBI [26, 27]. Markers of oxidative stress such as F2-Isoprostanes in cerebrospinal fluid have been associated with severe TBI [28, 29]. Although S100B has been most extensively studied and even incorporated into the Scandanavian Neurotrauma Committee guidelines [30], its relatively limited specificity may lead to overutilization of imaging studies.

Given the complexity of the intracranial injury response, it seems unlikely that any single biomarker would be sufficiently robust for use as a clinical diagnostic test. However, a peripheral, adjunctive blood-based biomarker panel consisting of multiple markers may flag patients with structural brain lesions who are in greater need of timely, appropriate management. This exploratory study was performed to identify biomarkers in the serum, which differentiate patients with uncomplicated mTBI from patients with complicated mTBI. Using a prospective study design, we believe that serum markers of inflammation, blood-brain barrier breakdown, neuronal injury, and oxidative stress collected within 24 hours of the trauma can be useful in differentiating patients who present with uncomplicated versus complicated mTBI. We also evaluated demographic and clinical data for their capacity to differentiate complicated and uncomplicated mTBI. Biomarkers that have received recent attention (e.g., S100B and brain derived neurotrophic factor) were also investigated.

## Methods

### Participants

This prospective cohort study was approved by the Duke University Institutional Review Board prior to data collection. Patients were recruited from the Duke University Medical Center Emergency Department between September 2007 and October 2008. Either the patient or the patient’s legal representative was required to provide written informed consent prior to participation. Inclusion criteria involved recent history of head trauma within 24 hours; documented brain imaging (within 24 hours); blood drawn within 24 hours of injury; a Glasgow Coma Scale on presentation ≥ 14; and, at least one or more of the following clinical symptoms: new onset headache, altered mental status, dizziness, vomiting, blurred vision, amnesia of the injury, amnesia prior to the injury, or loss of consciousness. Female patients were excluded for hemoglobin < 12.5g/dL and male patients were excluded for hemoglobin < 13.5g/dL to not exacerbate anemia by phlebotomy not associated with current clinical care. The Hgb criteria were used as a conservative cutoff for determination of anemia and are required by the Duke IRB for research involving blood collection in the absence of clinical need. Additional exclusion criteria included untreated systolic blood pressure < 90 mmHg and untreated diastolic blood pressure < 50 mmHg. The systolic and diastolic blood pressure criteria were used to exclude patients with diffuse systemic injury and hypoperfusion that might unduly influence the biomarker assays, particularly markers of oxidative stress. Injury severity score was also calculated as a clinical marker of extra-cranial injury.

Patients were stratified into 2 groups: uncomplicated and complicated mild traumatic brain injury patients. Uncomplicated mTBI was diagnosed if the patient met all inclusion criteria above and had no injuries visualized by a non-contrast head CT scan. A diagnosis of complicated mTBI was made if the patient met inclusion criteria and had evidence of a new subdural hematoma, epidural hematoma, subarachnoid hemorrhage, intraparenchymal hemorrhage, cerebral edema, or arterial dissection on non-contrast head CT. All radiology studies were performed by a clinical neuroradiologist as part of standard clinical practice and was otherwise blinded to study conditions.

The degree of concussion and trauma was also graded by commonly used scores including the Cantu guidelines [31], the 1997 American Academy of Neurology (AAN) guidelines [32], and Injury Severity Scores [33]. According to the Cantu guidelines, grade I concussions are associated with no loss of consciousness and less than 30 minutes of post-traumatic amnesia; grade II are associated with loss of consciousness for less than 5 minutes or amnesia between 30 minutes and 24 hours; and, grade III are associated with loss of consciousness greater than 5 minutes or amnesia longer than 24 hours [31]. The American Academy of Neurology guidelines stipulate that a grade I concussion is defined by no loss of consciousness and confusion less than 15 minutes; grade II involves symptoms lasting longer than 15 minutes; and, grade III involves loss of consciousness [32]. Finally, the Injury Severity Score (ISS) assesses trauma severity and is prognostic. It classifies injuries to six broad regions (head & neck; face; chest; abdomen; extremities; and, external). Injuries to each region are scored on a 6-point scale with a 6 indicating a mortal wound. The three highest scores (1–5) are squared and summed to create the ISS [33].

### Procedures and measurements

A blood sample of up to 10 milliliters by venipuncture, an existing peripheral IV, or an existing central line was obtained from each eligible patient who consented for the study within 24 hours of head injury. The samples were centrifuged within one hour of collection. Next, the supernatant was frozen at -80 degrees Celsius. These samples were then sent to Astute Medical, Inc. where tests were run on serum to measure the concentration of each of the 87 potential biomarkers. Procedures for these assays are described in the appendix. The biomarker panel was determined *a priori* and included constituents known to be associated with neuronal injury, inflammation, oxidative stress, gliosis, and vascular compromise [34]. A list of the potential biomarkers is presented in Supplementary S1 and S2 Tables. The electronic medical records of each patient were searched to extract clinical, demographic, laboratory, and radiographic data. In order to minimize bias, all investigators were blinded to the results.

### Statistical analyses

Statistical analyses were performed using SPSS v23 (IBM, Armonk, NY) and STATA v14.1 (reference). Descriptive statistics were calculated for demographic and clinical parameters for patients with complicated and uncomplicated mTBI. Means were compared between groups by Student’s t-test for continuous variables and counts (proportions) were compared by Pearson χ^2^ tests for categorical variables. Two-tailed hypotheses were used to analyze group differences on clinical and demographic variables (alpha = 0.05/2 = 0.025). A group effect was considered significant where p ≤ 0.025. Clinical and demographic data are presented in Table 1.

**Table 1:** 

Characteristic	CT Negative (N = 79)	CT Positive (N = 13)	P-value
Age (mean, SD)	45.8 (21.6)	48.2 (25.5)	0.36
Males (count, % of group)	52 (65.8%)	9 (69.2%)	0.41
Race			0.42
White	51 (64.6%)	8 (61.5%)	
Black + Other	28 (35.4%)	5 (38.5%)	
Mode of Trauma			0.22
Fall	28 (35.4%)	5 (38.5%)	
MVA	42 (53.2%)	8 (61.5%)	
Blunt object	9 (11.4%)	0 (0.00%)	
Time from injury to blood draw	6.6 (5.4)	9.9 (5.5)	0.02*
Radiographic Diagnosis^1^			
Subdural hematoma	-	3 (23.1%)	
Epidural hematoma	-	1 (7.7%)	
Subarachnoid hemorrhage	-	4 (30.8%)	
Intraparenchymal hemorrhage	-	3 (23.1%)	
Cerebral edema	-	1 (7.7%)	
Arterial dissection	-	1 (7.7%)	
Symptoms			
Headache	52 (65.8%)	10 (76.9%)	0.21
Altered mental status	14 (17.7%)	2 (15.4%)	0.42
Dizziness	8 (10.1%)	1 (7.7%)	0.39
Vomiting	4 (5.1%)	0 (0.0%)	0.2
Blurred vision	3 (3.8%)	0 (0.0%)	0.24
Amnesia of Injury	26 (32.9%)	7 (53.8%)	0.07
Amnesia of time prior	2 (2.5%)	2 (15.4%)	0.018*
Loss of consciousness	50 (63.3%)	10 (76.9%)	0.17
AAN	2.4 (0.84)	2.8 (0.60)	0.04
Cantu	1.7 (0.6)	2.0 (0.58)	0.06
Injury Severity Score	5.1 (5.8)	13.4 (9.2)	0.004*
Mini Mental Status Exam Score^2^	27.8 (3.8)	26.1 (2.5)	0.14

*Significance determined using a 2-tailed distribution (alpha/2 = 0.025).

^1^Diagnoses are not mutually exclusive.

^2^Available only for a limited set of subjects (CT− = 37, CT+ = 7)

Biomarkers were subjected to a two-step process: 1) biomarker selection, followed by 2) model development. The methods and criteria for this process follow from Hosmer and Lemeshow [35]. During the selection process, each biomarker was subjected to Student’s t-test to determine if the biomarker differed between groups (CT+ vs. CT−) using an *a priori* p ≤ 0.20 to minimize type II error. Biomarkers which were significantly different between groups were then tested for multicollinearity using the variance inflation factor (VIF ≤ 2.5). Those biomarkers meeting both criteria were retained for the model-building step.

The markers retained in the selection step above were then utilized to build two statistical models with the goal of effectively differentiating the CT- and CT+ groups. The first model was intended to be a broadly inclusive model while the second was intended to be a more parsimonious model. Variables were pared down by stepwise forward selection using the likelihood ratio criterion. The broad model was developed using p = 0.15 and p = 0.20 for entry and removal (respectively). The parsimonious model was developed using more stringent entry and removal criteria (p = 0.10 and p = 0.15, respectively). In both cases, clinical variables found to significantly differentiate groups were entered as covariates on step 1 of the regression. STATA was used to test the linearity of each variable in the logit using the multivariable fractional polynomial (MFP) procedure. The overall discriminative capacity of the model was ascertained by the concordance index or c-statistic, which captures the area under the curve (AUC) of the receiver operating curve (ROC). Sensitivities, specificities, positive predictive values, and negative predictive values were calculated at an outcome probability of 15 percent, which reflects the prevalence of an acute lesion on CT among patients who present with mTBI with a GCS of 15 [36]. Given the physiological complexity of these biomarkers, the derived biomarker model was limited to main effects. No interaction terms were investigated.

A rule of thumb in modeling that has been adopted widely in Cox and logistic regressions is that there should be a minimum of 10 events per predictor variable (EPV) based on 2 simulation studies [37, 38]. Results were particularly susceptible to bias and increased variability at an EPV less than 5. However, this threshold may be too conservative in several contexts including when validation methods are used. In a scenario of 5 EPV, the bootstrapped confidence intervals were more conservative than the Wald confidence intervals, often with coverage greater than 95 percent [39]. Thus, the rule of 10 EPV may be relaxed if validation is performed. The final models acquired by multivariate logistic regression were internally validated by bootstrapping the parameter estimates and c-statistic by generating 50 balanced re-samples.

## Results

### Characteristics of study subjects

A total of 110 mild TBI patients who met inclusion criteria were enrolled in this study. Seventeen of these patients had a positive head CT scan (CT^+^) and 93 patients had negative head CT scans (CT^−^). Three cases were eliminated for incomplete data and 15 cases were eliminated due to artifacts in the biomarker data. The final sample contained 92 cases: 79 CT^−^ and 13 CT^+^. Table 1 contains demographic, radiographic, and clinical information about patients in each group. The mean age of CT^−^ patients was slightly less than the mean age of CT^+^ patients, however they were not statistically different (45.8±21.6, 48.2±25.5, respectively). The patients were predominantly male and white in both groups with no proportional statistical difference between groups. The average latency between trauma and blood draw was 6.6 hours for CT^−^ patients and 9.9 hours for CT^+^ patients (TIME, p = 0.02). With respect to mechanism of trauma, there were no significant differences in the proportion of patients having fallen, been in a motor vehicle accident, or suffering blunt force trauma. The majority of patients with positive CT scans had radiographic evidence of subdural hematomas, subarachnoid hemorrhage, or intraparenchymal hemorrhage. In terms of symptoms, CT^+^ patients were more likely than CT^−^ patients to experience amnesia for time prior to the injury (API; 15.4% vs. 2.5%, p = 0.018) and there was a trend toward CT^+^ patients being more likely than CT^−^ patients to experience amnesia for the injury itself (53.8% vs. 32.9%, p = 0.07). There were no proportional differences in frequency of headache, altered mental status, dizziness, vomiting, blurred vision, or loss of consciousness (all p ≥ 0.17). The AAN, Cantu, and Mini Mental Status Exam (MMSE) scores were slightly higher among CT-positive patients but did not reach statistical significance. However, the Injury Severity Score (ISS) was significantly higher in CT^+^ patients than for CT^−^ patients (ISS, p = 0.004).

The three variables found to differ between groups (TIME, API, and ISS) were examined further using logistic regression. The model created using these three variables was statistically significant (χ^2^(3) = 19.43, p≤0.001) with goodness of fit preserved (Hosmer & Lemeshow, p = 0.53). While ISS and API were statistically significant in the model, TIME was not. Linearity of ISS and API were tested using the multivariable fractional polynomial procedure in STATA. API was found to be linear in the logit while ISS required transformation to ISS^-1^ to become linear. Following transformation, logistic regression using API and ISS^-1^ produced a significant model (χ^2^(3) = 25.74, p≤0.001) with goodness of fit preserved (Hosmer & Lemeshow, p = 0.998). For all subsequent analyses, API and ISS^-1^ were used as covariates in building and testing logistic models. Use of these covariates served to equate the CT+ and CT- groups in terms of injury severity (as measured by the presence of amnesia for time prior to the injury) as well as extra-cranial injury (as measured by the ISS).

### Demographic and clinical predictors

Logistic regression was used to test whether a demographic model consisting of age, sex, and race was predictive of imaging status (CT^+^ vs. CT^−^). Age was treated as a continuous variable while sex and race were treated as categorical. The regression was completed three times using different criterion groups for race. The first regression included age, sex and race where the group “white” was the race criterion. In the second and third regressions, “black” and “other” (Hispanic, Asian, Native American, Pacific Islander) served as the criterion group for race. None of the three demographic models were statistically significant above and beyond the covariates alone (all p ≥ 0.79). Moreover, none of these demographic variables were significant as individual variables in any of the three models (all p ≥ 0.43).

Based on their clinical utility, we analyzed the AAN and Cantu scores in a similar regression model. As noted with the demographic variables above, these clinical variables added nothing to the model above and beyond the covariates alone (p = 0.55). Moreover, neither AAN or Cantu scores were individually significant within the model (p = 0.85 and p = 0.55, respectively).

### Biomarker model development and evaluation

Student’s t-tests were performed to determine which serum biomarkers differed as a function of group (CT^+^ vs. CT^−^). Among the 87 markers assayed, 32 markers were statistically significant with p ≤ 0.20. Following evaluation for multicollinearity (VIF ≤ 2.5), only 12 variables remained in the pool of potential biomarkers. Results of the t-tests and multicollinearity tests are presented in the supplementary S1 Table. Means and standard errors for each biomarker in the CT^+^ and CT^−^ groups are found in supplementary S2 Table.

These 12 biomarkers were then used to build a broad multivariate logistic model, which was pared down by stepwise forward selection using the likelihood ratio criterion with p = 0.15 for entry and p = 0.2 for removal (Hosmer & Lemeshow, [35]). The transformed injury severity score (ISS^-1^) and amnesia for time prior to the accident (API) were entered as covariates on step one of the regression. The forward selection procedures were performed on step 2 of the regression. The broad model consisted of 6 biomarkers including MMP-2 (univariate AUC 0.616), CRP (univariate AUC 0.698), CKBB (univariate AUC 0.714), hFABP (univariate AUC 0.599), GM-CSF (univariate AUC 0.432) and MDA-LDL (univariate AUC 0.497). These 6 biomarkers resulted in a statistically significant model independent of the covariates (χ^2^(6) = 26.12, p≤0.001). The overall c-statistic for this 6-marker model (including the covariates) was 0.975 with Hosmer and Lemeshow Goodness of Fit preserved (p = 1.0). The Pearson correlations among these 6 variables ranged from -0.23 to 0.49. The test of linearity using MFP revealed all 6 variables were linear in the logit. This 6-variable model (including the covariates) was then subjected to internal validation by bootstrapping (n = 50 resamples) which yielded a bootstrapped c-statistic of 0.874.

**Fig 1:**
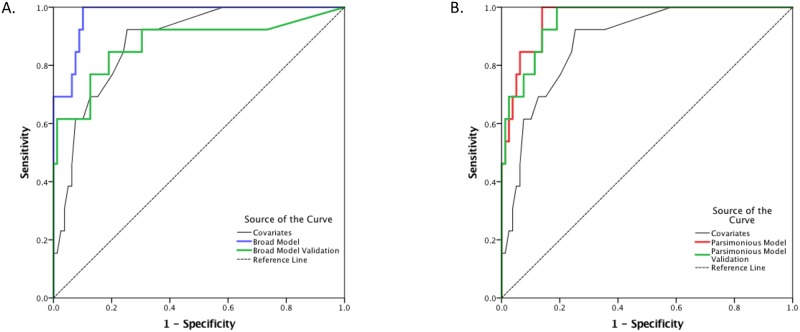
Receiver Operating Characteristic curves (ROC). ROC representing models used for discriminating complicated (CT+) versus uncomplicated (CT-) mTBI. Panel A: Broad Model (blue line, AUC = 0.975); Validated Broad Model (green line, AUC = 0.874); Covariates alone (black line, AUC = 0.882). Panel B: Parsimonious Model (red line, AUC = 0.964); Validated Parsimonious Model (green line, AUC = 0.956); Covariates alone (black line, AUC = 0.882).

**Fig 2:**
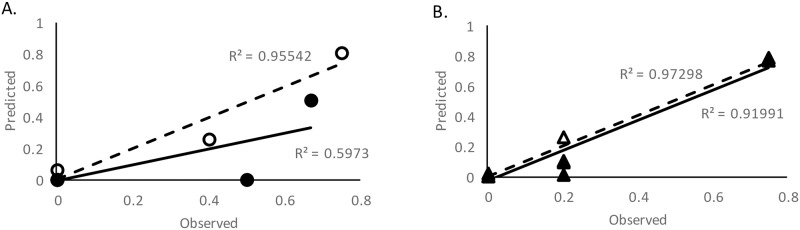
Calibration curves. Observed versus predicted proportion of complicated mild TBI outcome. Panel A: Broad Model (open circles) and corresponding validation (closed circles). Panel B: Parsimonious Model (open triangles) and corresponding validation (closed triangles). Figure also includes linear best-fit lines (model = dashed line, validation = solid line) and R^2^ values. (n = 92).

**Fig 3:**
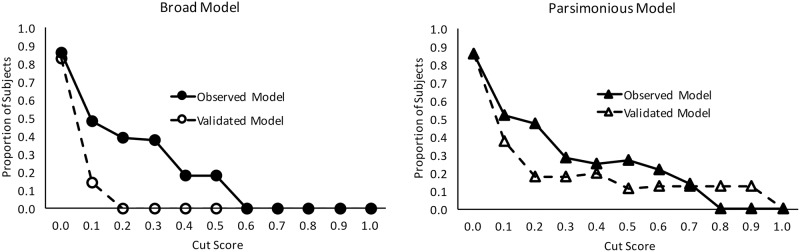
Predictive capacity. Observed versus validated proportion of patients who have a negative CT compared to the predicted probability values for each participant by the broad model (Panel A) and the parsimonious model (Panel B)

The 12 biomarkers identified in the selection process were also used to build a more parsimonious model using identical procedures, except more stringent criteria were used for entry (p = 0.10) and removal (p = 0.15). This parsimonious model consisted of the following: MMP-2 (univariate AUC 0.616), CRP (univariate AUC 0.698), and CKBB (univariate AUC 0.714). These 3 biomarkers (including the covariates) yielded a c-statistic (0.964) similar to that observed for the broad model (0.975). Hosmer and Lemeshow Goodness of Fit was preserved (p = 0.99). This 3 biomarker model was statistically significant independent of the covariates (χ^2^(3) = 19.03, p≤0.001). Intercorrelations between these 3 variables ranged from -0.23 to 0.18. All 3 of these variables were found to be linear in the logit according to MFP. Unlike the broad model described above, this parsimonious model retained most of its discriminative power following internal validation yielding a bootstrapped c-statistic of 0.956. ROC curves for both models are presented in Fig 1. Sensitivities, specificities, positive and negative predictive value are presented in Table 2. Fig 2 depicts the calibration curves produced by plotting the observed versus predicted probability of each CT+ study subject using each of the four models presented (broad model, validated broad model, parsimonious model, and validated parsimonious model). Fig 3 demonstrates the proportion of patients with negative CT scan findings among those with a certain predictive probability value generated by the broad and parsimonious models. This suggests that by predictive probability up to 0.2, the number of patients misidentified for CT decreases from 0.9 to 0 by the observed, broad model. This is in contrast to the parsimonious model where the cutoff of significance occurred at 0.8.

**Table 2:** 

Model	AUC	Sensitivity	Specificity	PPV	NPV
1. Covariates	0.882	84.6	75.9	36.7	96.8
2. Broad Model	0.975	92.3	89.9	60.0	98.6
Model Validation	0.874	69.2	97.5	81.8	95.1
3. Parsimonious Model	0.964	84.6	87.3	52.4	97.2
Model Validation	0.956	84.6	87.3	52.4	97.2

As a point of comparison, S100B and BDNF were also examined using logistic regression in a parallel set of procedures. However, no additional variance in CT status was accounted for by adding S100B (χ^2^(1) = 0.00, p = 0.99) or BDNF (χ^2^(1) = 3.58, p = 0.06) to the covariates on the second step of the regression. In addition, neither S100B nor BDNF were statistically significant as individual contributors to the model including the covariates (p = 0.99 and p = 0.18, respectively).

## Discussion

The primary goal of this study was to identify a panel of biomarkers that could accurately differentiate uncomplicated mTBI (those without evidence of intracranial bleeding) from complicated mTBI (those with evidence of intracranial bleeding). A blood based biomarker assay could provide a quick and minimally invasive process by which patients with complicated mTBI could be triaged to receive the necessary imaging to verify their extent of injury. Such an assay would have immediate clinical potential in military combat zones and those civilian contexts in which imaging tools were not readily available (e.g., pre-collegiate and collegiate athletics). Such assays would also have the potential benefit of lower cost, rapid analysis, and reduced exposure to radiation involved in unnecessary CT scans.

To that end, we screened a panel of 87 potential biomarkers from 110 patients admitted to the Duke University Medical Center Emergency Department who had sustained a mTBI within the last 24 hours, as assessed by GCS and clinical symptoms. These patients underwent CT imaging and blood draw within 24 hours of the incident. Individual biomarkers were assessed for significance and the 21 of those retained were then utilized to build 2 multivariate logistic models. One model was intended to be broad and more encompassing while the second was intended to be more parsimonious. The former was found to consist of 6 biomarkers while the latter was revealed to consist of only 3 biomarkers. Both models performed well and retained much of their predictive value following validation. We discuss below the parsimonious model due to its relative simplicity and improved predictive value.

The 3-biomarker model included CRP, MMP-2, and CKBB, which predicted the log-odds of a positive CT. The overall c-statistic for the 3-biomarker model was 0.975. The negative predictive value of the model was 97.2, which is comparable to the negative predictive value of 94.7 with troponin I for acute myocardial infarction [40]. In terms of the individual biomarkers, CRP is a sensitive but nonspecific biomarker of systemic inflammation in response to a variety of pro-inflammatory conditions, including infection, trauma, surgery, burns, tissue infarction, advanced cancer, and chronic inflammatory conditions [41]. CRP has previously been found to be a predictive biomarker of secondary pathologies associated with TBI [42]. Multiple studies have demonstrated an association between elevated CRP levels post-TBI and ongoing psychological dysfunction and cognitive impairment [43–45]. CRP has also been shown to have significant prognostic and diagnostic value in other conditions, including coronary heart disease, MI and ischemic stroke. CRP has been shown to predict the risk of coronary heart disease (CHD) events in men and women independently of traditional risk factors [46–48]. These studies also demonstrate that moderate elevations in CRP concentration that fall within the traditionally accepted ‘normal’ range have also been associated with myocardial infarction and ischemic stroke in prospective studies. Thus, our finding that CRP is a significant prognostic factor in determining the severity of a TBI is in line with a strong body of evidence that CRP is an important diagnostic and prognostic tool for a variety of inflammatory conditions, including TBIs.

The next biomarker, 72 kDa type IV collagenase, also known as matrix metalloproteinase-2 or MMP-2, is a member of a family of zinc-dependent proteases particularly involved in extracellular matrix remodeling. MMP-2 and MMP-9 are the most abundantly expressed proteins of the family in the brain and are thought to result from production and secretion by infiltrating inflammatory cells, neural cells, and endothelial cells [49]. The hyperactivation of metalloproteinases has been associated with cell death and neurogenesis depending on the stage of injury, though the mechanism of the latter has yet to be elucidated. MMP-2 is upregulated in the setting of transient focal cerebral ischemia in rat brains due to apoptosis as well as phagocytosis by macrophages [50]. One study demonstrated significant increase in MMP-2 levels at days 1, 3, and 5 after injury to the brain in rat models as well [51]. Thus, MMP-2 appears to be an acute marker of apoptosis and subsequent inflammatory response.

CKBB is an isoform of creatine-kinase found in the CNS. This enzyme catalyzes the transfer of phosphate groups from ATP to creatine phosphate, thus playing a role in energy transfer in tissues with large demands of energy such as the brain. CKBB is also present in significant quantities in the large intestine and prostate, although the concentration of the enzyme in these organs is one-third to one-fourth that seen in brain tissue [52]. Within the CNS, CKBB is located in astrocytes, and thus is released whenever there is brain tissue injury. Serum levels are initially elevated in acute trauma but return to baseline normal levels quickly. Serum levels of CKBB have been found elevated in various brain injury settings, including after cardiac arrest or subarachnoid hemorrhage [53, 54]. Coplin et al found the level of CKBB predicted unfavorable outcomes after subarachnoid hemorrhage [53]. Studies of severe and mTBI have found correlation between the severity of the injury and the serum level of CKBB [55–57]. Bakay and Ward observed a weak correlation between CKBB and brain injury severity as indexed by the GCS, but concluded that serum enzymatic determinations had an inadequate sensitivity and specificity for use as an indicator of neurologic trauma [58]. Other studies found that patients that CKBB failed to predict intracranial injury that was found on CT scan [59, 60]. However, several more recent studies have found CKBB does significantly correlate with brain injury after blast-induced trauma and severe trauma in proteomics analyses of CSF [61, 62]. Although previous studies suggest that CKBB concentration in serum is a poor single factor predictor of brain injury, our model offers the novel possibility that CKBB in sum with other biomarkers is a significant marker of brain injury.

S100B, a traditional biomarker of brain injury, was not found to significantly predict mild TBI versus no TBI. In the Scandinavian literature, there has been a study demonstrating the sensitivity of S100B to predict significant intracranial pathology up to 100% but specificity of only 28% [63]. Our method of employing area under the curve analysis was critical in ensuring that our panel of markers would maintain sensitivity and optimize specificity for intracranial pathology. A high negative predictive value was also preserved with the panel. These test characteristics lend themselves well to appropriate utilization of imaging.

Although we attempted to survey a broad array of potential biomarkers across several domains, including astrocytic activation, blood brain barrier dysfunction, chemokine activation, inflammatory response, oxidative stress, and neuronal injury, there are some biomarkers that were necessarily excluded from each category. Glial fibrillary acidic protein (GFAP) is one example. We selected several markers of astrocyte activation including 72 kDa type IV collagenase (MMP-2), alpha 2 macroglobulin, creatine kinase B-type (CKBB), fibronectin, leukemia inhibitory factor, and protein S100B. We believed at the time that these would offer superior sensitivity and specificity. More importantly, our goal was to focus primarily on chemokine and inflammatory markers which accounted for 63 of the 87 biomarkers studied. In retrospect, GFAP may have been a valuable contributor to the overarching model. Recent literature suggests that it may be a viable candidate biomarker and may be more relevant in many trauma settings than S100B [64, 65].

Development of a biomarker panel and its clinical implementation can be complicated. The data included in this study were collected within 24 hours of the incident. However, much remains unknown about the way in which mTBI pathophysiology evolves in the minutes and hours after injury. Our work did not account for the exact time post-injury that the imaging was performed and the blood samples were collected. Furthermore, our mean time from injury to sample collection was 6.6 hours in patients without positive imaging and 9.9 hours otherwise. It is interesting that the panel was predictive at such a length of time after injury, however, its predictive power needs to be determined acutely as well. In clinical situations, treatment and triage decisions are made at several time points post-injury. Future studies will need to carefully consider how the accuracy of the biomarker panel might change as a function time post injury.

Sample size was also a concern, especially among the CT positive patients. While the number of patients was appropriate for the stated goals of this study, the clinical utility of such a biomarker panel should be further evaluated using a large sample, prospective study across multiple medical centers. However, it is important to note that the proportion of CT positive patients within our sample (17 out of 110) is in keeping with percentages observed elsewhere (e.g., Smits et al., 2005). A consequence of the small sample size is that our study may have been biased toward patients with more extra cranial injury and/or more severe mTBI. The ISS (a measure of both intracranial and extra cranial injury) was significantly higher in our CT positive group than the CT negative group. Moreover, a higher proportion of the CT positive group reported amnesia for time prior to the injury (retrograde amnesia). This raises the possibility that our peripheral blood-based biomarker panel may have been sensitive to the extra-cranial injury or more severe mTBI, independent of radiographic evidence. However, the circumstance was addressed by using the ISS score and amnesia as a covariate in the development of these predictive models. The net result was to effectively equate the CT positive and CT negative groups. Finally, the ultimate utility of a biomarker assay to detect complicated mTBI rests on its relatively low cost and the rapidity with which results can be obtained. Beyond the practical identification of the specific biomarkers, there is much yet to be done on the technical aspects of producing cost effective and timely assay devices.

Our approach offers a strategy for assessing the role of multiple blood-based biomarkers in identifying which mTBI patients are likely to demonstrate intra-cranial injury using CT scan. The benefit of any such assay is two-fold. It can serve as a valuable tool for determining triage and transportation of those patients at highest risk for intra-cranial injury in situations where imaging technologies are not immediately available. In addition, it can be indispensable in reducing the number of unnecessary CT scans where the imaging technology is readily available. Such benefits are established by different data points [66]. The utility of an assay or algorithmic decision rule in determining triage priorities or the need for transportation to high level trauma centers is generally a function of sensitivity. The utility of an assay or algorithmic decision rule in reducing cost and radiation exposure associated with unnecessary imaging studies is generally a function of specificity. In this regard, our biomarker assay as well as the decision rules most frequently used (e.g., CCTHR, NOC, NEXUS-II) are generally equivalent, routinely reaching sensitivities of 100% and only rarely below 90% [6, 67–69]. However, there is far greater variability among these decision rules when it comes to specificity [66, 70]. One recent study [71] indicates that the CCTHR yielded a specificity (65%) and NPV commensurate with that reported in our study. Unfortunately, that study has been met with some criticism due to widely variable sensitivities and specificities when validated in cohorts in other countries [66]. In fact, it is possible that cultural and medical practice variables account for a significant percentage of the variability between studies of these decision rules.

Regardless of the absolute sensitivity, specificity, and negative predictive value of these decision rules, they are not as frequently used as might have been expected. Data from urban trauma centers suggest that as many as 10–45% of the head CT studies conducted on ED admissions with minor head trauma were unnecessary [72, 73], depending on the decision rules used. In yet another study, only 58% of physicians surveyed who ordered CT imaging in mTBI cases (GCS = 15) cited “guidelines” as their rationale for doing so [7]. Interestingly, it has been suggested that greater confidence in imaging studies and the practice of defensive medicine may play a key role in the under-utilization of these decision rules [73]. Indeed, among primary care physicians surveyed regarding advanced imaging techniques (including CT), 88% felt that imaging afforded greater confidence in diagnoses; 85% felt that patient care would be diminished without imaging; and, 80% felt that it reduced the time to initiating appropriate treatment [74]. While emergency department physicians and primary care physicians differ in many respects, it is clear that there is some truth in the idea that “seeing is believing”. That is, physicians may choose to order imaging studies because they have greater confidence in the images they see, than the numbers and metrics yielded by abstract and impersonal decision rules. It is conceivable, that results of a multi-marker, blood-based assay will result in fewer unnecessary imaging studies because physicians will have greater confidence in medical results they can “see”.

In the end, our results support the need for further investigation of multi-marker, blood-based biomarker assays to identify those mTBI patients that should undergo radiographic studies to rule out intra cranial injury. Our results demonstrate that CRP, CKBB, and MMP-2, provide excellent sensitivity, specificity, and negative predictive value that can effectively identify those individuals at greatest risk of intra cranial injury and simultaneously reduce the need for unnecessary imaging. We should note, our biomarker models do not produce demonstrably better sensitivity, specificity, and negative predictive values than those associated with the covariates alone (API and ISS^-1^). However, use of these variables would constitute yet another decision rule for determining which patients should receive imaging and which should not. It is unlikely that these variables or a decision rule derived from them would be implemented any more frequently than those discussed above. The very nature of a blood-based medical test may elicit the confidence needed to avoid ordering unnecessary imaging. Such confidence may be absent when using strictly algorithmic decision rules.

## Appendix A

Astute Medical, Inc. protocols for measuring biomarker levels depending on the readability of each biomarker’s antibody by the two instruments:

### Luminex protocol

A pre-wet filter plate were filled with 300 uL/well wash buffer, then vacuum and blotted dry. A bead mixure was made with all appropriate concentrated bead conjugates and added at a volume of 25 uL/well to the filter plate, which was washed and then vacuum dried. Bead diluent was added at a volume of 25 uL/well to all wells. Next, 25 uL/well of assay buffer was added to sample wells, background wells, and internal control wells. A prepared serum matrix was added to the background, standard, and kit control wells at a volume of 25 uL/well, with a total volume up to this step of 75 uL/well. The plate was then incubated for 2 hours at 600 rpm at room temperature. Next, it underwent 3 washes with 300 uL of wash buffer and was vacuum and blot dried. Next, detection antibodies were added at a volume of 25 uL/well. The plate was again incubated at 600 rpm. Next, 25 uL/well of streptavidin-RPE was added and then plate was incubated at 600 rpm for 30 minutes. Another 3 series of washes were conducted with 300 uL of wash buffer and then dried by vacuum and blotting. Next, 100 uL/well sheath fluid was added and the plate was re-incubated at 600 rpm for 5 minutes. The assay plate was then read on a Luminex instrument.

### Meso Scale Discovery(MSD) protocol

Diluent 2 was added at a volume of 25 uL/well to the MSD plate, which was then incubated at 600 rpm for 30 minutes at room temperature. Standards, internal controls, and samples were added to appropriate wells at 25 uL/well. The total volume per well was 50 uL/well after this step. The plate was then incubated at 600 rpm for 2 hours and washed with 300 uL of TECAN hydroflex 3 times. The plate was inverted and tapped on absorbent material 3 times to remove excess was buffer from the wells. The concentrated detection antibody mixture was diluted to 1x and added to each well at a volume of 25 uL/well. The plate was again incubated at 600 rpm for 2 hours, then washed 3 times with 300 uL of TECAN hydroflex. The plate was inverted and tapped on absorbent material at least 3 times. Next, 150 uL/well of 2x MSD Read Buffer was added. The assay plate was then read on the MSD instrument.

All assays were performed according to the manufacturer’s instructions and protocol.

## Supporting information

S1 Fig Graphical representation of steps used for creating biomarker models. Eighty-seven biomarkers from 6 categories were tested for differences between CT+ and CT- patients (p≤0.2). Those 32 biomarkers were assessed for multicollinearity (VIF≤2.5). The 12 remaining biomarkers were subjected to forward logistic regression using amnesia for time prior to the injury and the injury severity score as covariates. Two models were derived: a broad model and a parsimonious model. The broad model was developed using p = 0.15 for entry and p = 0.2 for removal while the parsimonious model was developed using p = 0.10 for entry and p = 0.15 for removal.
(TIF)

S1 Table Original pool of biomarkers (n = 87). Data included provides outcome information for the biomarker selection process: pairwise comparison and VIF analyses.
(PDF)

S2 Table Biomarker descriptive data. Data included provide means and standard deviations for each the original biomarkers broken out by group (CT+ vs. CT-).
(PDF)
