# Supplementary figures and images for: A blood-based biomarker panel to risk-stratify mild traumatic brain injury
Source: PLoS One. 2017 Mar 29;12(3):e0173798. doi: 10.1371/journal.pone.0173798 (PMC5371303; doi:10.1371/journal.pone.0173798)

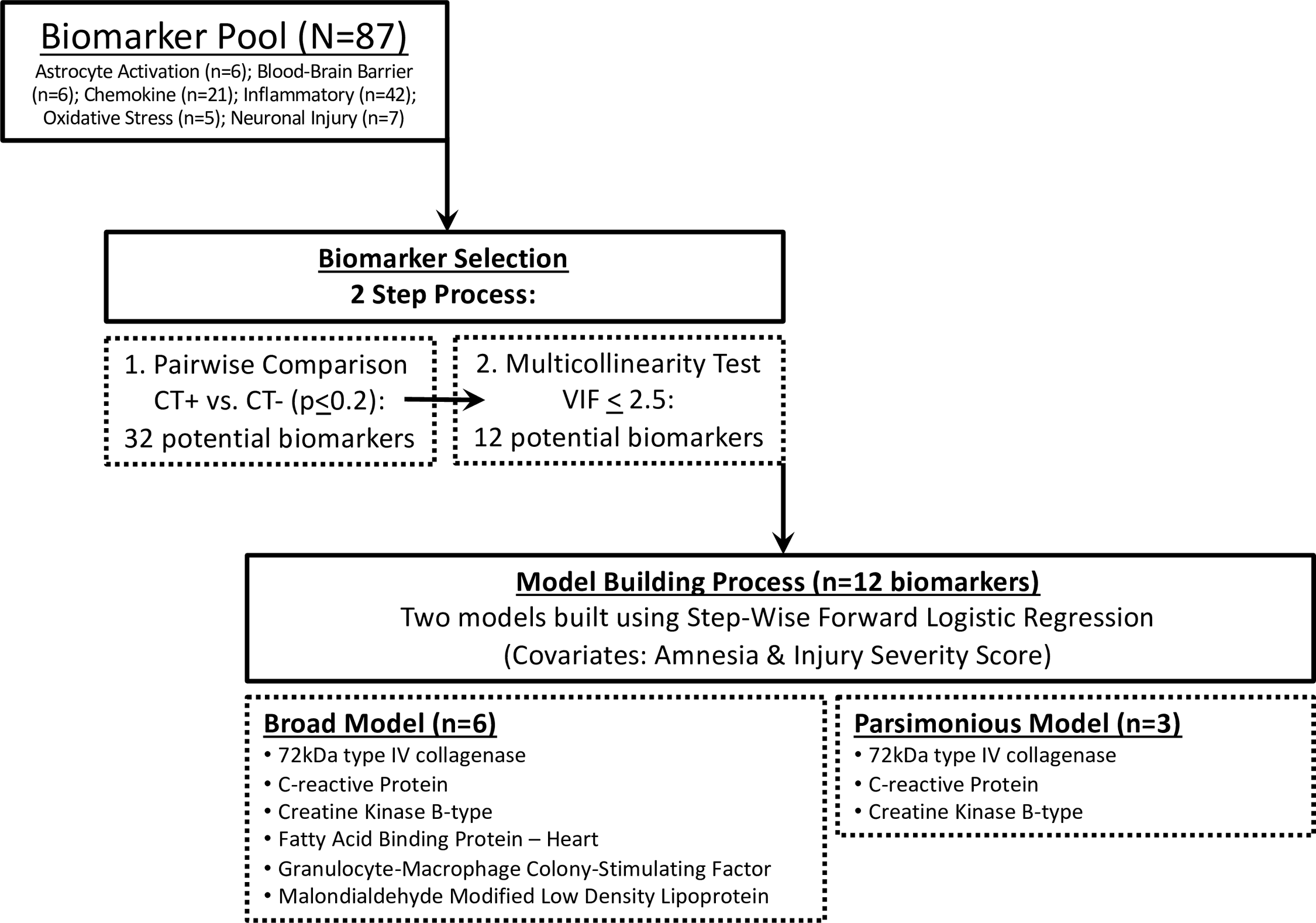

Supplement: S1 Fig — Eighty-seven biomarkers from 6 categories were tested for differences between CT+ and CT- patients (p≤0.2). Those 32 biomarkers were assessed for multicollinearity (VIF≤2.5). The 12 remaining biomarkers were subjected to forward logistic regression using amnesia for time prior to the injury and the injury severity score as covariates. Two models were derived: a broad model and a parsimonious model. The broad model was developed using p = 0.15 for entry and p = 0.2 for removal while the parsimonious model was developed using p = 0.10 for entry and p = 0.15 for removal. (TIF) [file pone.0173798.s001.tif]
