# Supplementary material for: A blood-based biomarker panel to risk-stratify mild traumatic brain injury
Source: PLoS One. 2017 Mar 29;12(3):e0173798. doi: 10.1371/journal.pone.0173798 (PMC5371303; doi:10.1371/journal.pone.0173798)
Supplement: S1 Table — Data included provides outcome information for the biomarker selection process: pairwise comparison and VIF analyses. (PDF) [file pone.0173798.s002.pdf]

Supplemental Table 1.

Biomarker	p	VIF	M	Biomarker	p	VIF	M
**72 kDa type IV collagenase (v2)**	**0.08**	**2.4***	B/P	Interleukin-12	0.24		
Alpha-2 macroglobulin	0.48			Interleukin-13	0.30		
Alpha-2-HS-glycoprotein	0.46			**Interleukin-15**	**0.19**	**16.9**	
**Amyloid beta A4 protein (fragment AB40)**	**0.13**	**5.5**		Interleukin-17A	0.34		
**Amyloid beta A4 protein (fragment AB42)**	**0.13**	**4.5**		Interleukin-2	0.24		
Apolipoprotein(a)	0.48			Interleukin-2 receptor alpha chain (v2)	0.38		
**Brain-derived neurotrophic factor**	**0.09**	**23.8**		Interleukin-20	0.35		
Cathepsin D	0.48			Interleukin-21	0.40		
C-C motif chemokine 1	0.35			Interleukin-23	0.39		
C-C motif chemokine 13 (v1)	0.27			Interleukin-28A	0.34		
**C-C motif chemokine 13 (v2)**	**0.11**	**4.2**		**Interleukin-3**	**0.08**	**11.6**	
C-C motif chemokine 15	0.33			Interleukin-33	0.37		
C-C motif chemokine 17 (v1)	0.26			Interleukin-4 (v1)	0.23		
**C-C motif chemokine 17 (v2)**	**0.18**	**8.3**		Interleukin-6	0.35		
C-C motif chemokine 2	0.23			**Interleukin-7**	**0.20**	**26.0**	
C-C motif chemokine 21	0.49			**Interleukin-8 (v2)**	**0.05**	**31.8**	
**C-C motif chemokine 22**	**0.11**	**1.8**		**Interleukin-8 (v3)**	**0.05**	**31.7**	
C-C motif chemokine 24	0.42			Interleukin-9	0.30		
C-C motif chemokine 26	0.35			Kit ligand	0.34		
C-C motif chemokine 27	0.46			Leukemia inhibitory factor	0.36		
C-C motif chemokine 3	0.28			Lymphotoxin-alpha	0.27		
C-C motif chemokine 4	0.40			**Malondialdehyde-modified low-density** lipoprotein	**0.19**	**1.6***	B
**C-C motif chemokine 5**	**0.08**	**52.3**					
C-C motif chemokine 7	0.24			**Matrix metalloproteinase-9 (v2)**	**0.09**	**2.8**	
C-C motif chemokine 8	0.20			Mix of Growth-regulated alpha, beta, and gamma proteins	0.41		
**CD40 ligand**	**0.08**	**11.1**					
**C-reactive protein**	**0.07**	**1.6***	B/P	Myeloperoxidase	0.25		
**Creatine kinase B-type**	**0.03**	**2.2***	B/P	**Natriuretic peptides B**	**0.16**	**2.3**	
C-X-C motif chemokine 10	0.31			**Neural cell adhesion molecule 1**	**0.15**	**1.3**	
C-X-C motif chemokine 13	0.30			**Oxidized low-density lipoprotein receptor 1**	**0.20**	**3.0**	
**C-X-C motif chemokine 5**	**0.19**	**6.8**		**Platelet-derived growth factor subunit A** (dimer)	**0.15**	**32.9**	
Eotaxin	0.36						
**Fatty acid-binding protein, heart**	**0.16**	**1.4***	B	**Platelet-derived growth factor subunit B;** AB/BB (dimer)	**0.12**	**51.4**	
Fibrinogen (v2)	0.49						
Fibronectin	0.45			**Pro-epidermal growth factor (v2)**	**0.13**	**7.9**	
Fms-related tyrosine kinase 3 ligand Flt-3	0.32			Pro-interleukin-16	0.31		
Glial cell line-derived neurotrophic factor	0.30			Protein S100-B	0.30		
**Granulocyte-macrophage colony-stim factor**	**0.16**	**1.9***	B	**Serum amyloid P-component**	**0.05**	**1.6**	
**Haptoglobin**	**0.06**	**2.2**		Stromelysin-1 (v2)	0.29		
**Heparin-binding growth factor 2 (v2)**	**0.18**	**3.4**		Thrombomodulin (v1)	0.26		
Intercellular adhesion molecule 1	0.30			Thrombopoietin	0.35		
Interferon alpha-2	0.30			Thymic stromal lymphopoietin	0.33		
Interferon gamma	0.31			**Tumor necrosis factor**	**0.10**	**3.0**	
Interleukin-1 alpha	0.38			Tumor necrosis factor ligand superfamily member 10	0.23		
Interleukin-1 beta	0.33						
Interleukin-1 receptor antagonist protein	0.33			**von Willebrand Factor**	**0.11**	**1.8**	
Interleukin-10	0.31						

Note: VIF=Variance Inflation Factor; M=Model Inclusion (B=Broad, P=Parsimonious)
