# Supplementary material for: A blood-based biomarker panel to risk-stratify mild traumatic brain injury
Source: PLoS One. 2017 Mar 29;12(3):e0173798. doi: 10.1371/journal.pone.0173798 (PMC5371303; doi:10.1371/journal.pone.0173798)
Supplement: S2 Table — Data included provide means and standard deviations for each the original biomarkers broken out by group (CT+ vs. CT-). (PDF) [file pone.0173798.s003.pdf]

Supplementary Table 2

Biomarker (Category & Name)	CT	N	Mean	SEM
Astrocytic Activation				
72 kDa type IV collagenase (v2)	0	79	313233.71	10094.21
	1	13	351561.87	24185.37
Alpha-2 macroglobulin	0	79	1009.86	42.07
	1	13	1004.99	73.26
Creatine kinase B-type	0	79	37.27	2.35
	1	13	64.08	13.22
Fibronectin	0	79	295140.80	8484.65
	1	13	292506.35	21389.62
Leukemia inhibitory factor	0	79	126.74	120.41
	1	11	6.63	4.89
Protein S100-B	0	78	3.69	0.64
	1	13	4.56	1.57
Blood Brain Barrier				
Platelet-derived growth factor subunit A (dimer)	0	79	1559.87	194.76
	1	13	1057.13	207.08
Platelet-derived growth factor subunit B; AB/BB (dimer)	0	79	15541.29	2747.45
	1	13	7336.68	1695.75
Stromelysin-1 (v2)	0	79	7350.26	549.29
	1	13	8152.60	1310.20
Thrombomodulin (v1)	0	79	1875.25	96.27
	1	13	2058.75	368.03
Thrombopoietin	0	79	2513.52	1604.24
	1	11	810.40	806.82
von Willebrand Factor	0	79	44058.66	2809.80
	1	13	53206.09	5516.92
Chemokine				
C-C motif chemokine 1	0	79	6.32	3.80
	1	11	2.39	0.70
C-C motif chemokine 13 (v1)	0	79	52.04	21.70
	1	11	16.18	6.78
C-C motif chemokine 13 (v2)	0	79	1496.63	113.38
	1	13	1143.84	180.15
C-C motif chemokine 15	0	79	2316.74	176.73
	1	11	2102.08	432.32
C-C motif chemokine 17 (v1)	0	79	40.37	6.12
	1	11	29.85	5.03

Supplementary Table 2, Continued

Biomarker (Category & Name)	CT	N	Mean	SEM
C-C motif chemokine 17 (v2)	0	79	184.34	25.28
	1	13	126.88	19.28
C-C motif chemokine 2	0	79	1493.77	114.13
	1	13	1733.15	386.11
C-C motif chemokine 21	0	79	832.51	357.60
	1	11	855.49	359.04
C-C motif chemokine 22	0	79	878.97	40.19
	1	13	1012.90	105.74
C-C motif chemokine 24	0	79	434.23	43.43
	1	11	459.33	81.99
C-C motif chemokine 26	0	79	92.66	80.57
	1	11	6.00	3.63
C-C motif chemokine 27	0	79	524.08	24.76
	1	11	532.43	85.10
C-C motif chemokine 3	0	79	22.02	13.98
	1	13	1.34	0.70
C-C motif chemokine 4	0	79	110.59	32.90
	1	13	89.84	29.42
C-C motif chemokine 5	0	79	58999.65	8796.11
	1	13	27778.33	5526.00
C-C motif chemokine 7	0	79	20.67	5.96
	1	13	9.95	5.69
C-C motif chemokine 8	0	79	29.91	6.13
	1	11	16.15	3.06
C-X-C motif chemokine 10	0	79	1018.58	183.96
	1	13	784.48	185.13
C-X-C motif chemokine 13	0	79	68.84	17.95
	1	11	43.40	6.17
C-X-C motif chemokine 5	0	79	512.65	68.36
	1	11	348.59	124.46
Eotaxin	0	79	911.15	48.64
	1	13	865.39	96.04
Inflammatory				
Alpha-2-HS-glycoprotein	0	79	471240.36	10727.37
	1	13	474120.73	22726.58
CD40 ligand	0	79	2371.46	479.05
	1	13	672.73	177.39
C-reactive protein	0	79	6361.67	1050.37
	1	13	13573.41	4570.67

Supplementary Table 2, Continued

Biomarker (Category & Name)	CT	N	Mean	SEM
Fibrinogen (v2)	0	79	1403.72	59.24
	1	13	1409.11	168.91
Fms-related tyrosine kinase 3 ligand Flt-3	0	79	4.12	1.98
	1	13	1.75	1.50
Granulocyte-macrophage colony-stimulating factor	0	79	0.47	0.11
	1	13	2.25	1.71
Haptoglobin	0	79	336902.47	53786.04
	1	13	166404.89	91994.89
Intercellular adhesion molecule 1	0	79	181121.90	6103.88
	1	13	173012.84	8377.68
Interferon alpha-2	0	79	56.64	12.24
	1	13	40.87	7.67
Interferon gamma	0	79	1.17	0.72
	1	13	0.26	0.10
Interleukin-1 alpha	0	79	28.71	13.57
	1	13	18.26	15.57
Interleukin-1 beta	0	79	0.29	0.17
	1	13	0.11	0.05
Interleukin-1 receptor antagonist protein	0	79	59.64	22.53
	1	13	35.17	17.61
Interleukin-10	0	79	8.39	2.60
	1	13	5.10	1.89
Interleukin-12	0	79	13.05	6.97
	1	13	0.78	0.23
Interleukin-13	0	79	9.31	6.31
	1	13	1.23	0.71
Interleukin-15	0	79	7.05	3.00
	1	13	0.51	0.24
Interleukin-17A	0	79	14.39	7.54
	1	13	6.62	2.13
Interleukin-2	0	79	0.22	0.03
	1	13	0.28	0.08
Interleukin-2 receptor alpha chain (v2)	0	79	40.75	25.92
	1	13	20.52	14.83
Interleukin-20	0	79	426.22	346.05
	1	11	66.10	34.41
Interleukin-21	0	79	23.50	18.35
	1	11	36.81	36.43
Interleukin-23	0	79	2717.92	1666.36
	1	11	1438.61	1430.24

Supplementary Table 2, Continued

Biomarker (Category & Name)	CT	N	Mean	SEM
Interleukin-28A	0	79	164.29	142.34
	1	11	5.67	4.66
Interleukin-3	0	79	1.59	0.28
	1	13	0.59	0.24
Interleukin-33	0	79	221.20	163.23
	1	11	77.55	77.33
Interleukin-4 (v1)	0	79	4.30	2.31
	1	13	0.06	0.05
Interleukin-6	0	79	14.43	3.57
	1	13	17.90	3.46
Interleukin-7	0	79	4.63	1.68
	1	13	1.06	0.57
Interleukin-8 (v2)	0	79	132.26	18.54
	1	13	281.62	81.96
Interleukin-8 (v3)	0	79	5.29	0.75
	1	13	11.51	3.45
Interleukin-9	0	79	3.58	2.52
	1	13	0.23	0.12
Lymphotoxin-alpha	0	79	15.87	10.20
	1	13	0.38	0.36
Matrix metalloproteinase-9 (v2)	0	79	235031.30	25362.21
	1	13	378018.03	97822.26
Myeloperoxidase	0	78	938.79	36.60
	1	13	1006.30	94.68
Neural cell adhesion molecule 1	0	79	296602.07	10229.95
	1	13	324322.70	18880.36
Oxidized low-density lipoprotein receptor 1	0	79	0.24	0.03
	1	13	0.30	0.05
Pro-epidermal growth factor (v2)	0	79	31.45	6.83
	1	13	12.54	3.65
Pro-interleukin-16	0	79	144.50	52.54
	1	11	72.91	16.23
Thymic stromal lymphopoietin	0	79	345.03	237.55
	1	11	55.98	54.54
Tumor necrosis factor	0	79	5.47	0.36
	1	13	6.82	1.19
Tumor necrosis factor ligand superfamily member 10	0	79	43.82	15.84
	1	11	11.86	2.74

Supplementary Table 2, Continued

Biomarker (Category & Name)	CT	N	Mean	SEM
Oxidative Stress				
Amyloid beta A4 protein (fragment AB40; Amyloid Beta 40)	0	79	64.84	3.73
	1	13	77.38	15.23
Amyloid beta A4 protein (fragment AB42; Amyloid Beta 42)	0	79	32.54	1.64
	1	13	27.80	2.57
Apolipoprotein(a)	0	79	92814.19	11897.85
	1	13	94565.43	15866.39
Fatty acid-binding protein, heart	0	79	1.24	0.39
	1	13	8.43	6.84
Malondialdehyde-modified low-density lipoprotein	0	79	580.42	192.08
	1	13	161.65	38.70
Neuronal Injury				
Brain-derived neurotrophic factor	0	79	3363.81	475.78
	1	13	1749.10	345.09
Cathepsin D	0	79	171448.62	14238.51
	1	13	169165.47	19739.43
Glial cell line-derived neurotrophic factor	0	78	2.30	1.27
	1	13	0.64	0.25
Heparin-binding growth factor 2 (v2)	0	79	143.13	35.38
	1	13	63.69	13.50
Kit ligand	0	79	83.47	53.99
	1	11	21.90	6.65
Natriuretic peptides B	0	79	886.43	313.58
	1	13	2460.24	1500.54
Serum amyloid P-component	0	79	9700.67	361.13
	1	13	8096.09	1041.52
